# Case Report: imaging features of anaplastic lymphoma kinase-rearranged renal cell carcinoma with a novel *DCTN1::ALK* fusion

**DOI:** 10.3389/fonc.2026.1732867

**Published:** 2026-03-10

**Authors:** Fei Sang, Weiwei Zhang

**Affiliations:** 1 Department of Radiology, Peking University Shenzhen Hospital, Shenzhen, Guangdong, China; 2 Department of Ultrasound, Peking University Shenzhen Hospital, Shenzhen, Guangdong, China

**Keywords:** anaplastic lymphoma kinase, case report, computed tomography, *dctn1::ALK* fusion, imaging features, renal cell carcinoma

## Abstract

Anaplastic lymphoma kinase (ALK)-rearranged renal cell carcinoma (ALK-RCC) is an extremely rare subtype of RCC, accounting for less than 1% of all cases. Its computed tomography (CT) features remain poorly characterized, hindering preoperative diagnosis. We report the case of a 34-year-old female who presented with painless gross hematuria. Abdominal ultrasound revealed a well-circumscribed, hyperechoic heterogeneous mass in the upper pole of the right kidney. Contrast-enhanced CT demonstrated a solitary, medullary-based hypodense mass with multiple punctate and patchy calcifications and mild heterogeneous enhancement. The patient underwent laparoscopic nephron-sparing surgery with complete resection. Histopathology showed papillary architecture with ISUP/WHO grade 2 atypical cells. Immunohistochemistry was diffusely positive for ALK (clone D5F3). Fluorescence in situ hybridization confirmed ALK rearrangement, and next-generation sequencing identified a novel DCTN1::ALK fusion—the first report of such a fusion in ALK-RCC. Follow-up showed no evidence of recurrence. This case highlights distinctive CT features of ALK-RCC that may raise suspicion in young patients and guide molecular testing. The identification of the *DCTN1::ALK* fusion expands the molecular landscape of ALK-RCC and supports the potential utility of ALK inhibitors.

## 1 Introduction

In the 2022 WHO renal tumor classification, anaplastic lymphoma kinase (ALK)-rearranged renal cell carcinoma (ALK-RCC) was formally recognized as a distinct and uncommon subtype, accounting for less than 1% of all RCC cases (1). This tumor is defined by rearrangements of the ALK gene, which drive oncogenesis through constitutive activation of the ALK tyrosine kinase and downstream signaling pathways such as PI3K/AKT/mTOR and MAPK/ERK (2). Clinically, ALK-RCC predominantly affects young individuals (mean age 29.6 years) with no gender predilection. The most common presenting symptom is painless gross hematuria, followed by abdominal discomfort or incidental detection (3).

Despite its rarity, ALK-RCC holds clinical significance due to emerging evidence that ALK inhibitors—such as entrectinib and alectinib—may induce durable responses in advanced cases (4, 5). However, diagnosis remains challenging due to overlapping clinical and morphological features with common RCC subtypes (e.g., papillary RCC, clear cell RCC) (3). Imaging (especially computed tomography (CT)) is critical for preoperative evaluation (6, 7), but few studies have described ALK-RCC’s CT features (8, 9), limiting early recognition.

Prior ALK-RCC studies identified fusion partners (VCL, TPM3, EML4, STRN) (2, 10), but no DCTN1::ALK fusion has been reported in RCC. To address gaps in imaging and molecular characterization, this report aims to (1): detail CT features of histopathologically and molecularly confirmed ALK-RCC (2); report the first DCTN1::ALK fusion in RCC (3); provide insights into the diagnosis and management of this rare subtype.

## 2 Case presentation

A 34-year-old female presented with painless gross hematuria. Initial evaluation at an external hospital revealed unremarkable urinalysis results; however, abdominal ultrasound performed as a first-line screening tool showed a 67×55 mm well-circumscribed, hyperechoic heterogeneous mass in the upper pole of the right kidney. The mass contained irregular internal hypoechoic areas, exhibited no internal blood flow signals, and caused compression of the upper renal calyx. The patient was subsequently referred to our hospital for further evaluation. She reported no other symptoms such as abdominal pain, weight loss, or fever. Her past medical, surgical, and family histories were non-contributory.

Routine laboratory tests were unremarkable. Complete blood count, coagulation function, urinalysis (no proteinuria/glycosuria), renal function (serum creatinine 58 μmol/L, blood urea nitrogen 3.4 mmol/L), and serum tumor markers (CA125, CEA, AFP) were all within normal limits.

**Figure 1:**
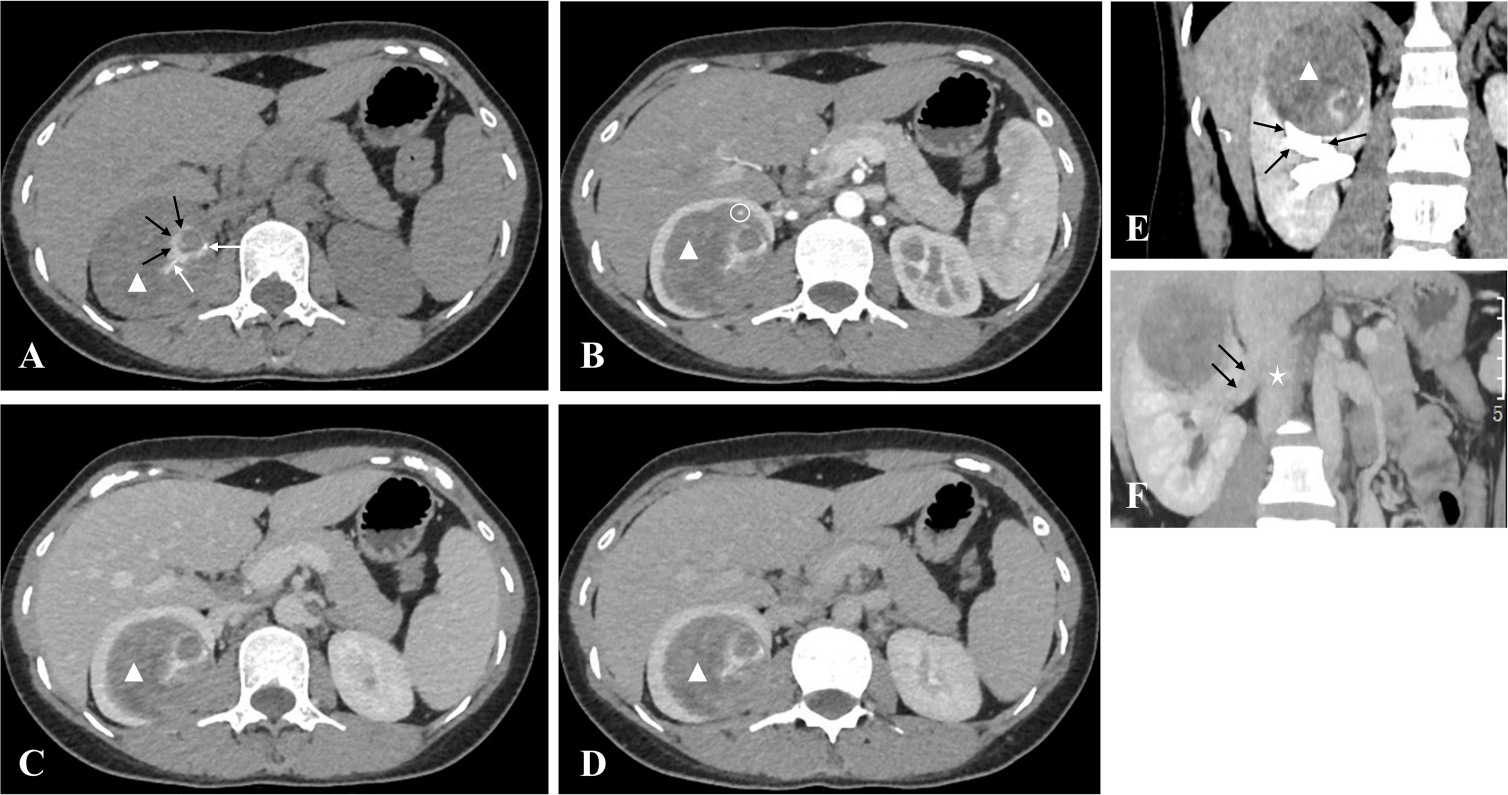
Non-contrast and contrast-enhanced abdominal computed tomography (CT) images of the right renal anaplastic lymphoma kinase-rearranged renal cell carcinoma**. (A)** Axial non-contrast CT shows a round right renal mass (white triangle) located in the medulla, measuring 54×55×65 mm with heterogeneous hypodensity (mean 46 HU). Multiple punctate and patchy calcifications (white arrow) and flocculent slightly hyperdense areas (black arrow) are visible within the mass. **(B)** Axial corticomedullary-phase CT demonstrates mild heterogeneous enhancement of the mass (mean 52 HU; enhancement ratio 1.13 vs. non-contrast, white triangle), with a feeding artery branch (white circle) originating from the right renal artery. **(C)** Axial nephrographic-phase CT shows increased enhancement of the mass (mean 58 HU; enhancement ratio 1.26 vs. non-contrast, white triangle). **(D)** Axial excretory-phase CT shows further mild enhancement of the mass (mean 61 HU; enhancement ratio 1.33 vs. non-contrast, white triangle). **(E)** Coronal excretory-phase CT confirms the medullary location of the mass (white triangle) and its compression of the upper renal calyces (black arrow). **(F)** Oblique coronal maximum intensity projection image (nephrographic phase) shows no filling defects in the right renal vein (black arrow) or inferior vena cava (white star).

Non-contrast and contrast-enhanced CT were performed upon admission. CT values were measured in areas of prominent enhancement using regions of interest (200 mm², twice per phase) by two radiologists with at least five years of experience in abdominal imaging. Interobserver agreement was excellent (ICC = 0.986). Non-contrast CT revealed a 54 × 55 × 65 mm round, well-circumscribed mass in the right renal medulla, causing compression of the upper renal calyx (**Figures 1A, E**). The mass appeared heterogeneously hypodense, containing multiple punctate, patchy, and curvilinear calcifications (**Figure 1A**), as well as flocculent slightly hyperdense areas (**Figure 1A**). Contrast-enhanced CT demonstrated mild heterogeneous enhancement across all phases (**Figures 1B–D**), with no filling defects in the renal vein or inferior vena cava (**Figure 1F**) and no retroperitoneal lymphadenopathy.

Given the patient’s preference and TNM stage I classification (no evidence of invasion or metastasis), laparoscopic nephron-sparing surgery (NSS) was performed to preserve renal function. Complete resection was achieved, and the patient had an uneventful postoperative recovery.

Gross pathological examination of the resected specimen revealed a 6 cm × 5.5 cm × 5 cm well-circumscribed tumor with a grayish-yellow to brown variegated cut surface and a soft-to-firm consistency. No gross necrosis or hemorrhage was observed. Histopathology confirmed negative surgical margins (0.1–0.2 cm tumor distance from margin). Microscopic evaluation showed well-defined borders and expansile growth, with atypical cells arranged in papillary and micropapillary architectures. The cells had round-to-oval nuclei of moderate size, abundant eosinophilic granular cytoplasm, and focal hemorrhage. Foamy histiocytes and cholesterol clefts were also present. Notably, while calcifications were a prominent radiological feature of the tumor, they were not identified in the sampled pathological sections, likely owing to the heterogeneous intratumoral distribution of the calcified foci. No necrosis, sarcomatoid or rhabdoid differentiation, lymphovascular invasion, or perineural involvement was seen. The tumor was classified as ISUP/WHO grade 2 based on nuclear atypia.

Immunohistochemistry showed positive staining for CK7, PAX8, Vimentin, ALK (clone D5F3), and P504S, and negative staining for TFE3, CD10, CD117, and CAIX. Retained nuclear expression was observed for MLH1, PMS2, MSH2, MSH6, succinate dehydrogenase B, and fumarate hydratase.

Fluorescence in situ hybridization was performed with the Vysis ALK Break Apart Probe (Guangzhou KingMed Diagnostic Center, Guangzhou, China). 71 of 200 analyzed cells showed split red (3′ALK) and green (5′ALK) signals, confirming ALK rearrangement (35.5% positive rate). Targeted DNA/RNA next-generation sequencing (Jinan KingMed Diagnostic Center, Jinan, China; 51 RCC-related genes) identified a DCTN1::ALK fusion (20.55%, 216 matching reads)—the first report in ALK-RCC. Additional validation (RT-PCR, 5′-RACE/Sanger sequencing) was unfeasible due to insufficient residual formalin-fixed paraffin-embedded tissue.

The patient did not receive adjuvant ALK inhibitor therapy, as no FDA-approved regimens for ALK-RCC were available at the time of treatment initiation (11). Follow-up imaging (non-contrast CT at 1 and 9 months, abdominal ultrasound at 5 months) showed no local recurrence or distant metastasis. The patient remained asymptomatic.

## 3 Discussion

### 3.1 Clinical and imaging significance of ALK-RCC

This case details ALK-RCC’s CT features, which align with limited prior data (8, 9) and add specificity for young patients. Key CT findings—medullary location, hypodensity, multiple calcifications, mild heterogeneous enhancement—differentiate ALK-RCC from common subtypes, guiding preoperative strategies.

Clear cell RCC, the most prevalent subtype, typically shows a “wash-in, wash-out” enhancement pattern (12), which was absent in this case. Papillary RCC, another common subtype, shares mild enhancement but lacks medullary predilection and calcifications (6). Renal medullary carcinoma, a rare and aggressive tumor, overlaps with ALK-RCC in terms of young patient age and medullary location. However, it rarely presents with calcifications and often shows advanced features such as renal sinus invasion at diagnosis (9).

Notably, ALK-RCC affects young individuals (mean age 29.6 years (3)), while common RCC subtypes occur in adults over 60 years (13). Thus, a medullary-based hypodense renal mass with calcifications and mild heterogeneous enhancement in patients under 40 years should prompt ALK-RCC suspicion and further testing (ALK immunohistochemistry and molecular testing) (14). Early identification is critical, as ALK inhibitors have been shown to improve survival outcomes in advanced ALK-RCC cases (4, 5).

### 3.2 Novelty of *DCTN1::ALK* fusion in RCC

To our knowledge, this is the first DCTN1::ALK fusion report in ALK-RCC. A systematic literature search (PubMed, Embase, Web of Science; query: “(DCTN1-ALK OR DCTN1 ALK OR DCTN1::ALK OR DCTN1/ALK) AND (renal cell carcinoma OR RCC OR kidney cancer)”; no date restrictions) confirmed no prior RCC reports.

*DCTN1::ALK* fusion has been described in other malignancies (lung adenocarcinoma, infant glioblastoma) (15). Its identification in RCC expands ALK-RCC’s molecular spectrum beyond known partners (*VCL, TPM3, EML4, STRN*) (2, 10). DCTN1 encodes a dynactin complex component critical for microtubule-based intracellular transport (16). In other cancers, dysregulation of DCTN1 has been associated with increased progression and poor prognosis (16), suggesting that *DCTN1::ALK* may confer unique biological properties in ALK-RCC. This hypothesis requires validation in larger cohorts.

Preclinical lung cancer studies show DCTN1::ALK fusion retains constitutive ALK kinase activity (15), which is the key oncogenic driver in ALK-rearranged tumors. This supports potential ALK inhibitor utility (e.g., alectinib, crizotinib) in DCTN1::ALK-positive ALK-RCC, given their efficacy in other ALK-rearranged malignancies (17). However, clinical data remain limited, and future trials are needed (11).

### 3.3 Treatment regimen

Our patient’s management (NSS without adjuvant ALK inhibitors) aligns with current localized ALK-RCC evidence. For localized disease, NSS is favored in eligible patients to preserve renal function. This approach is consistent with Elhassan et al. (8), who reported successful robotic-assisted NSS for ALK-RCC with no short-term recurrence, matching our 9-month recurrence-free follow-up. This reinforces NSS as safe for young stage I patients, where renal function preservation is prioritized.

In contrast, advanced ALK-RCC responds to ALK inhibitors. Pal et al. (5) documented durable partial responses to alectinib in two metastatic cases, and Kathuria-Prakash et al. (4) reported a partial response to alectinib in refractory metastatic disease. These reports validate the use of ALK inhibition for advanced ALK-RCC, including our *DCTN1::ALK* case, given that the fusion retains ALK kinase activity (15).

Notably, our patient received no adjuvant ALK inhibitors due to lack of FDA-approved adjuvant regimens (11). This gap was highlighted by Iannantuono et al.’s (11) systematic review, which noted adjuvant use remains investigational. Future studies should evaluate adjuvant ALK inhibitors for high-risk localized cases (e.g., positive margins, high grade) using metastatic efficacy data.

### 3.4 Limitations

This study has three limitations: (1) *DCTN1::ALK* fusion was confirmed via fluorescence *in situ* hybridization and next-generation sequencing (consistent results), but multi-platform validation (RT-PCR/5′-RACE/Sanger sequencing) is ideal for novel fusions. This was not feasible due to insufficient residual formalin-fixed paraffin-embedded tissue. (2) High-quality gross, microscopic, and fluorescence *in situ* hybridization images could not be included. While surgery was performed at our institution, we lacked pathologist support to retrieve and prepare histopathologic slides for photography. Additionally, fluorescence *in situ* hybridization and sequencing images were unavailable from the third-party laboratory due to strict patient privacy regulations and institutional data-sharing policies. Detailed textual descriptions of all pathological and molecular findings are provided to compensate for this limitation. (3) Magnetic resonance imaging was not performed, which might have provided additional tissue composition insights for ALK-RCC characterization. However, ultrasound and CT provided sufficient information for tumor localization, characterization, and staging.

### 3.5 Patient perspective

The patient was actively involved in the treatment decision-making process. After being informed of the renal mass and its malignant potential, she expressed anxiety about both cancer progression and potential loss of renal function. She received detailed counseling on treatment options, including radical nephrectomy and nephron-sparing surgery, with explanations of the risks, benefits, and long-term quality of life implications. She prioritized renal function preservation and strongly preferred nephron-sparing surgery, provided that oncological safety could be assured. She was also informed about the rarity of ALK-RCC and the lack of established adjuvant therapy guidelines, which she understood and accepted. Postoperatively, she reported satisfaction with her recovery and the cosmetic outcome. During follow-up, she expressed relief at the absence of recurrence and emphasized the importance of regular monitoring. She also conveyed hope that the identification of the novel *DCTN1::ALK* fusion in her tumor might contribute to future research and benefit other patients. Written informed consent for publication was obtained.

## 4 Conclusion

This case reports the first DCTN1::ALK fusion in ALK-RCC and details its distinctive CT features including solitary, medullary-based hypodensity with calcifications and mild heterogeneous enhancement. These findings may serve as a diagnostic clue for ALK-RCC in young patients, facilitating early molecular testing and potential targeted therapy access. The DCTN1::ALK fusion expands ALK-RCC’s molecular landscape and supports ALK inhibitors as a potential therapeutic option. Future large-cohort studies and long-term follow-up are needed to validate DCTN1::ALK’s biological properties and ALK inhibitor efficacy in ALK-RCC.

## Data Availability

The original contributions presented in the study are included in the article/supplementary material. Further inquiries can be directed to the corresponding author.
